# UFBP1 Ameliorates Heat Stress-Induced Apoptosis via Mitochondria-Mediated Pathway in Bovine Mammary Epithelial Cells

**DOI:** 10.3390/ani15091233

**Published:** 2025-04-27

**Authors:** Yuan Li, Ran Yu, Shujing Tan, Yunlong Jiang, Longwei Sun, Manman Shen, Chuanjian Zhang, Kunlin Chen, Chengmin Li

**Affiliations:** 1 Jiangsu Key Laboratory of Sericultural and Animal Biotechnology, School of Biotechnology, Jiangsu University of Science and Technology, Zhenjiang 212100, China; liyuan981225@163.com (Y.L.); 18912250570@163.com (R.Y.); tanshujing2023@126.com (S.T.); 17787269727@163.com (Y.J.); sunlongwei163@163.com (L.S.); shenman2005@163.com (M.S.); 2 Key Laboratory of Silkworm and Mulberry Genetic Improvement, Ministry of Agriculture and Rural Affairs, The Sericultural Research Institute, Chinese Academy of Agricultural Sciences, Zhenjiang 212100, China; 3 Institute of Veterinary Immunology and Engineering, Jiangsu Academy of Agricultural Sciences, Nanjing 210014, China; zcj6717855@126.com; 4 Institute of Animal Science, Jiangsu Academy of Agricultural Sciences, Ministry of Agriculture and Rural Affairs, Nanjing 210014, China; chenkunlin@jaas.ac.cn

**Keywords:** heat stress, bovine mammary epithelial cells (BMECs), UFBP1, mitochondrial function, apoptosis, milk synthesis

## Abstract

Heat stress in dairy cows is aggravated by Global warming, which negatively affects their performance and health, especially high yielding cows are more susceptible to high temperature and humidity in summer. Besides increasing body temperature and reducing feed intake, heat stress also compromises mammary gland function by inducing apoptosis in bovine mammary epithelial cells (BMECs). UFBP1 (Ufm1-binding protein 1) serves as an essential component of ufmylation, is crucial for the preservation of cellular homeostasis. However, little is known about its contribution to heat stress-induced apoptosis in BMECs. Therefore, the present study aimed to elucidate the effect of UFBP1 on heat stress-induced apoptosis through knockdown and overexpression of UFBP1 in BMECs. The results showed that heat stress triggered cell apoptosis (increased apoptosis rate and Bax/Bcl-2 protein expression) and decreased the expression of genes associated with the production of milk fat and protein both in vivo and in vitro studies. Furthermore, UFBP1 silencing aggravated the high-temperature-induced cell damage, and overexpression of UFBP1 attenuated heat stress-induced mitochondrial dysfunction, as evidenced by increased mitochondrial membrane potential (MMP), ATP synthesis and NAD^+^/NADH ratio, as well as the reduced reactive oxygen species (ROS) generation. Importantly, the mitochondrial apoptosis pathway triggered by heat stress was blocked by UFBP1, as indicated by the reduced apoptosis rate and Bax/Bcl-2 protein expression. In addition, UFBP1 restored the expression of milk fat and protein-related genes in heat-stressed BMECs. In conclusion, these findings indicate that UFBP1 may serve as a promising therapeutic target for ameliorating heat stress in dairy cows, thereby providing novel theoretical insights into the mitigation of adverse thermal stress effects on livestock productivity.

## Simple Summary

Global warming exacerbates heat stress in dairy cows, leading to apoptosis and decreased secretion function in bovine mammary epithelial cells (BMECs). UFBP1 (Ufm1-binding protein 1) functions as a critical element in ufmylation, which is crucial for the maintenance of cellular homeostasis. In the present study, we explored the role of UFBP1 in heat stress-induced apoptosis in mammary epithelial cells. The results showed that UFBP1 significantly alleviated the increase in reactive oxygen species (ROS), mitochondrial impairment and cell apoptosis triggered by thermal stress in BMECs. Moreover, overexpression of UFBP1 restored the expression of genes involved in the biosynthesis of milk fat and protein. These results indicated that UFBP1 possessed potential therapeutic value for mitigating heat stress in dairy cows.

## 1. Introduction

The increased incidence of heat stress in livestock caused by global climate warming has negative effects on the performance and health of animals by disrupting body balance, stability and normal function. Dairy cattle, especially high productive dairy cows, are more vulnerable to summer heat stress, due to higher thermogenesis while producing milk [1,2]. The function of bovine mammary gland in milk synthesis and storage is undoubtedly threatened by high body temperature and heat stress-induced low feed intake, which brings a severe restriction on the development of global dairy industry [3,4]. Additionally, various in vitro studies show that bovine mammary epithelial cells (BMECs), the main functional units for milk production of mammary glands, are also affected by heat treatment and exhibit higher rates of programmed cell death when exposed to high temperatures [5,6,7].

Accumulating evidence has revealed that heat stress enhances reactive oxygen species (ROS) production, which can induce cellular apoptosis of BMECs, thereby contributing to decreases in milk production and quality in dairy cows [8,9,10]. The milk fat and protein synthesis efficiency is positively associated with milk quality. SREBP1 regulates milk fat synthesis by regulating genes like FASN and ACACA, while β-Casein (CSN2) participates in the formation of milk protein, thereby serving as indicators of milk fat and protein synthesis in BMECs [11,12,13]. Recent studies indicate that mitochondria are not only the main source of energy production but also participate in several other biological processes, including the ROS generation and cell apoptosis [14,15]. More specifically, mitochondrial dyshomeostasis elevates the intracellular levels of ROS and the translocation of mitochondrial substances such as cytochrome C (cyto-c) into cytoplasm, thus triggering caspase-3 activation and igniting apoptosis [16,17]. Therefore, exploring potential strategies to counteract the adverse effects of mitochondria-dependent apoptosis is particularly important in protecting dairy cows from heat stress.

Ufmylation, the post-translational modification by ubiquitin-like proteins (Ubls) is essential in multiple cellular processes, such as cell apoptosis, endoplasmic reticulum (ER) homeostasis regulation, and stress response [18,19,20]. Ufm1 binding protein 1 (UFBP1, also known as DDRGK1, C20orf116, and Dashurin) is a key component of ufmylation that can form a large multiprotein complex with other ufmylation components Ufl1, C53/LZAP and Ufm1 [21,22]. Recently, studies have reported that UFBP1 is indispensable in the maintenance of ER homoeostasis [22,23,24]. Loss of UFBP1 can cause a range of physiological and pathological responses, including ER stress, UPR activation, intestinal dyshomeostasis and hematopoietic dysfunction [25,26]. Furthermore, UFBP1 have a higher enrichment in secretory cells, which has been found to protect cells against ER stress-induced apoptosis [24,27]. Therefore, we hypothesized that UFBP1 could mediate the apoptosis of bovine mammary epithelial cells in response to heat stress. The objective of this study was to elucidate the effect of UFBP1 on heat stress-induced apoptosis by knocking down and overexpressing UFBP1 through the mitochondrial pathway in bovine mammary epithelial cells.

## 2. Materials and Methods

### 2.1. Animals and Sample Collection

The animal experiments involving dairy cows were approved by the Animal Ethics Committee of the Jiangsu University of Science and Technology (approval number: 20200306). A total of 6 Chinese Holstein cows (56 ± 2.4 months old, 135 ± 19 (DIM), *n* = 6) were selected for this experiment and randomly divided into two groups: the control group experiment was conducted during the cool spring of April, and the heat stress group was investigated during the hot summer of August, with dairy cows identified as experiencing heat stress based on a temperature and humidity index (THI) value > 80, respiratory rate > 80 breaths/min, and rectal temperature > 39.5 °C. The environmental conditions of both groups had been monitored and maintained in a stable state for more than three weeks preceding sample collection.

The mammary parenchyma was sampled from the dairy cows during experimental period using percutaneous needle biopsy. All biopsied cows were fasted for 12 h preoperatively and anesthetized by local injection of 2% lidocaine at the biopsy site, then 3–5 mammary tissue strips were obtained by a 14-gauge puncture needle, and the samples were immediately fixed in paraformaldehyde and frozen in liquid nitrogen.

### 2.2. Histology and Immunostaining

The hematoxylin-eosin (HE) and immunohistochemistry staining were performed as previously described [28]. Briefly, Breast tissues were fixed with 4% paraformaldehyde, paraffin-embedded and cut into 5 μm sections, and stained with HE to observe pathological changes. For immunohistochemical evaluation, the sections were incubated overnight with UFBP1 primary antibody (1:400, Proteintech, Chicago, IL, USA) and then for 1 h with goat anti-rabbit IgG secondary antibody. The levels of positive cells were used to assess the expression level of UFBP1. The TUNEL assay (Beyotime, Shanghai, China) to detect apoptotic cells was performed according to manufacturer’s instructions, and DAPI was used to visualize nuclei. Green fluorescence indicates apoptotic cells and blue fluorescence represents nucleus. Images were acquired by fluorescence microscopy (Olympus, Tokyo, Japan).

### 2.3. Cell Culture, Transfection, and Treatments

Bovine mammary epithelial cell lines were obtained from the Animal Biotechnology Laboratory of Jiangsu University of Science and Technology, which are characterized by y their cobblestone-like morphology and high proliferation activity, and serve as an in vitro model for studying lactation and mammary gland biology. Cells were cultured in DMEM/F12 medium (Gibco, Grand Island, NY, USA) supplemented with 10% fetal bovine serum (Gibco, Grand Island, NY, USA) and 100 U/mL of penicillin and streptomycin (Invitrogen, Carlsbad, CA, USA) in an atmosphere of 5% CO_2_ and 90% humidity at 37 °C. To determine the effect of heat exposure on BMECs, cells were cultured in an incubator at 43 °C for 1, 2, 3, 4, 5, and 6 h, respectively. Based on the expression of HSP70 and apoptosis-related genes, a heat stress model was established at 43 °C for 3 h.

The pcDNA3.1—UFBP1 overexpression vector was constructed by using pcDNA3.1 as a recombinant cyclisation vector. Small interfering RNAs (siRNAs) targeting UFBP1 were designed and synthesized by GenePharma (Shanghai, China). BMECs were transfected with UFBP1 overexpression plasmid and siRNA using Lipo8000™ Transfection Reagent (Beyotime, Shanghai, China) for 6 h following the manufacturer’s specifications. At the same time, a pcDNA3.1 empty vector and non-target siRNA were transfected as negative controls for the overexpression and knockdown assays, respectively. The UFBP1 siRNA sequence is 5′-CCUUGAGUCACAGCGUGAATT-3′, and the antisense sequence is 5′-UUCACGCUGUGACUCAAGGTT-3′, the NC sequence was 5′-UUCUCCGAACGUGUCACGUTT-3′, and the antisense sequence was 5′-ACGUGACACACGUUCGGAGAATT-3′, cells were collected after 48 h for subsequent experiments. The transfection efficiency was determined by Western blot. After 48 h of transfection, cells were treated at 43 °C for 3 h in an incubator.

### 2.4. Flow Cytometry to Detect Cell Apoptosis

Apoptosis in BMECs subjected to heat stress was assessed with the Annexin V-FITC Apoptosis Detection Kit (Beyotime, Beijing, China). Following the manufacturer’s protocol, cells were treated with Annexin V-FITC and PI, then incubated at room temperature for 25 min. The labeled cells were then measured by flow cytometry (BD Biosciences, Bedford, MA, USA), and the resulting data were analyzed using FlowJo software (Version9, Becton, Dickinson and Company, Franklin Lakes, NJ, USA).

### 2.5. Detection of Intracellular Reactive Oxygen Species

The level of intracellular reactive oxygen species (ROS) was evaluated using 10 mM DCFH-DA (Beyotime, Shanghai, China) as a specific fluorescent probe. After a 20 min incubation at 37 °C, cells were rinsed with PBS to eliminate unbound probes. Fluorescence imaging was performed immediately using an inverted microscope with excitation at 500 nm and emission detected at 525 nm (Olympus, Tokyo, Japan), and mean fluorescence intensity was quantified using ImageJ software (Version 1.53t, National Institutes of Health, Bethesda, MD, USA).

### 2.6. Detection of Cellular ATP Levels and the NAD^+^/NADH Ratio

After treating the cells at 43 °C for 3 h, the ATP levels in BMECs were measured using an ATP assay kit (Beyotime Beijing, China). Cells were collected and lysed, then centrifuged at 12,000 rpm at 4 °C for 15 min. The supernatants were assayed for ATP levels in accordance with the manufacturer’s instructions. The ratio of NAD^+^/NADH was measured using the NAD^+^/NADH assay kit (Beyotime Beijing, China). The levels of NADH_total_ and NADH in the assay were quantified using a microplate reader at 450 nm, and the NAD^+^ concentration calculated by subtracting NADH from total NADH.

### 2.7. Mitochondrial Membrane Potential Assays

The changes in mitochondrial membrane potential were evaluated with a JC-1 fluorescent probe (Beyotime, Shanghai, China). After incubation with JC-1 staining solution at 37 °C for 20 min, BMECs were washed twice using JC-1 (1×) buffer. Fluorescence in the cells was analyzed using microscopy, and the intensity was measured with ImageJ software (Version 1.53t, National Institutes of Health, Bethesda, MD, USA). The ratio of red fluorescence to green fluorescence was used to calculate the mitochondrial membrane potential.

### 2.8. Western Blot Analysis

Total protein from cultured BMECs and mammary gland tissue samples were lysed with a buffer containing immunoprecipitation reagent (RIPA), protease inhibitor and (PMSF). A total of 200 µL of lysates were added to the cells in six-well plates or 20–25 µg of bovine mammary tissue, and after centrifugation at 4 °C, the protein concentration of the supernatant was determined using the BCA Protein Assay Kit (Beyotime, Shanghai, China). From there, 20 μg protein/lane of the samples were resolved using SDS-PAGE and it was then transferred onto a polyvinylidene difluoride (PVDF) membrane using a semi-dry transfer apparatus (GenScript, Shanghai, China). The PVDF membrane was incubated with the following specific primary antibodies overnight at 4 °C after blocking with 5% skimmed milk: rabbit anti-UFBP1 (1:1000, Proteintech, 21445-1-AP, Chicago, IL, USA), rabbit anti-caspase3 (1:1000, Proteintech, 25128-1-AP, Chicago, IL, USA), rabbit anti-Bax (1:1000, 50599-2-Ig, Proteintech, 21445-1-AP, Chicago, IL, USA), rabbit anti-Bcl-2 (1:1000, A19693, ABclonal, Boston, MA, USA), rabbit anti-SREBP1 (1:1000, A15586, ABclonal, Boston, MA, USA), rabbit anti-CSN2 (1:1000, A12749, ABclonal, Boston, MA, USA), rabbit anti-FASN (1:1000, A0461, ABclonal, Boston, MA, USA), rabbit anti-HSP70 (1:1000, A12948, ABclonalA12948, ABclonal, Boston, MA, USA). Rabbit anti-β-Tubulin (1:4000, AP0064, Bioworld, Louis Park, MN, USA) and Rabbit anti-β-Actin (1:4000, GB15003-100, Servicebio, Wuhan, China) were used as reference proteins. The PVDF membrane was then washed with TBST and incubated with HRP-conjugated secondary antibody. Enhanced chemiluminescence (ECL) reagents (Pierce, Rockford, IL, USA) were used to visualize and detect the immunoreactive bands. Finally, the protein bands were scanned and calculated by ImageJ software (Version 1.53t, National Institutes of Health, Bethesda, MD, USA).

### 2.9. Statistical Analysis

Data are presented as mean ± SEM (Standard Error of the Mean), derived from a minimum of three independent experimental repetitions. Statistical analysis was conducted using GraphPad Prism 8.0 software (La Jolla, CA, USA). Differences among groups were assessed using one-way analysis of variance (ANOVA) followed by Tukey’s test. Significance levels were set at * *p* < 0.05, ** *p* < 0.01.

## 3. Results

### 3.1. Expression of UFBP1 in Heat-Stressed Mammary Gland and BMECs

**Figure 1:**
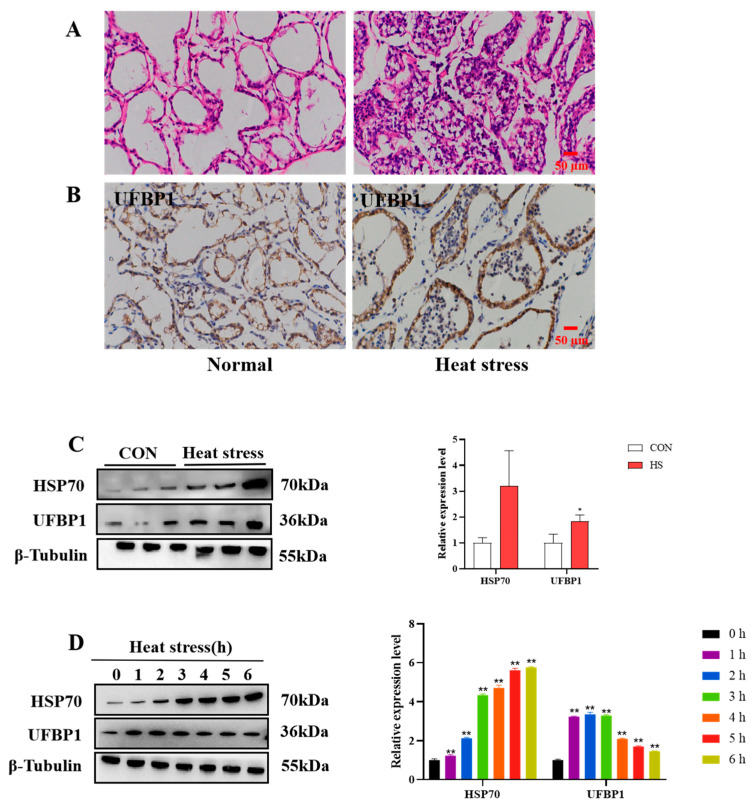
Expression of UFBP1 in the mammary gland and BMECs. (**A**) HE staining of mammary gland tissue in normal and heat-stressed dairy cows. (**B**) Immunohistochemical staining of mammary gland tissue with UFBP1 antibody. (**C**,**D**) Expression of UFBP1 and HSP70 in bovine mammary gland (**C**) and BMECs (**D**). Results are expressed as mean ± SEM from three independent replicates. * *p* < 0.05; ** *p* < 0.01.

In the current study, we first investigated the level of UFBP1 in mammary tissues and BMECs under heat stress conditions. HE staining results indicated that, in the normal group, the mammary gland exhibited regular arrangement and intact structures, while heat-stressed dairy cows presented disorganized alveolar structures, accompanied by atrophy and infiltration of inflammatory cells (Figure 1A). Immunohistochemical staining analysis showed that UFBP1 was upregulated to a greater extent in the mammary tissue of heat stressed cows compared to normal cows (Figure 1B). Furthermore, Western blot analysis showed that the level of heat shock protein HSP70 was significantly higher in heat-stressed cows compared with the normal group (Figure 1C). In addition, the level of UFBP1 was examined in BMECs after exposure to heat at 43 °C for different times (1, 2, 3, 4, 5 and 6 h). The results showed that the heat shock protein 70 (HSP70) level was significantly increased during heat treatment in a time-dependent manner, and UFBP1 expression showed a significant upregulation after heat exposure, peaking at 2 h post-treatment and then declining (Figure 1D).

### 3.2. Heat Stress Induces Apoptosis in Heat-Stressed Mammary Gland and BMECs

**Figure 2:**
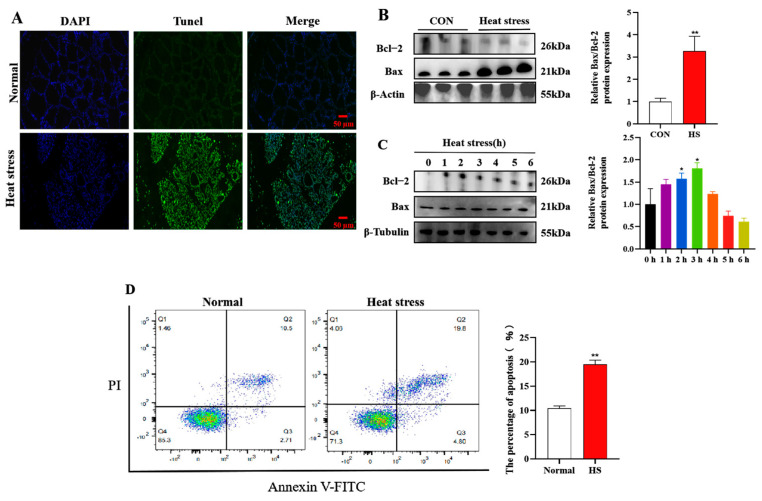
Effect of heat stress on apoptosis in heat-stressed mammary gland and BMECs. (**A**) TUNEL staining of mammary gland tissue. (**B**,**C**) Expression of apoptosis-related proteins in heat-exposed mammary gland tissue (**B**) and BMECs (**C**). (**D**) Flow cytometric analysis of apoptosis in BMECs. Results are expressed as mean ± SEM from three independent replicates. * *p* < 0.05; ** *p* < 0.01.

TUNEL analysis indicated that the amount of apoptotic cells in mammary tissue of heat-stressed dairy cows was markedly higher than that of the normal cows (Figure 2A). Meanwhile, Western blot analysis showed that for the apoptosis index, the Bax to Bcl-2 ratio was significantly increased in dairy cows under heat stress (Figure 2B). Moreover, we also explored how heat stress affected BMECs apoptosis. Results showed that the ratio of Bax to Bcl-2 protein was considerably elevated during 2 and 3 h of heat exposure (Figure 2C). Consistent with the Western blot results, flow cytometric analysis of apoptosis showed that the number of apoptotic cells was significantly higher after heat treatment at 43 °C for 2 h than that of the normal group (Figure 2D).

### 3.3. Heat Stress Inhibits Milk Fat and Protein Synthesis-Related Gene Expression

**Figure 3:**
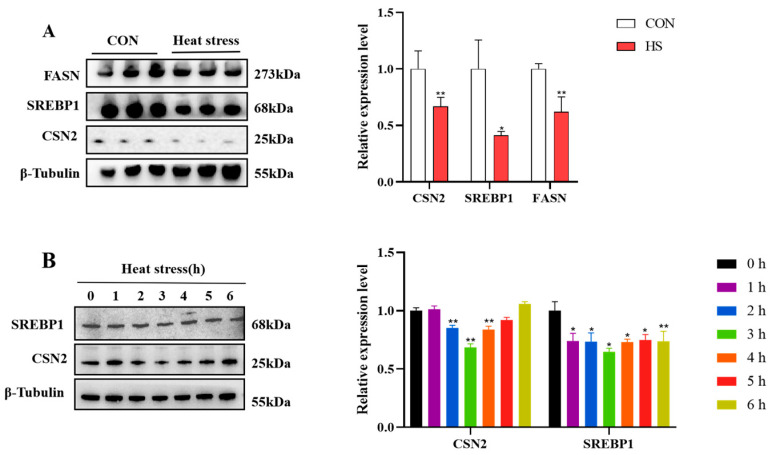
Protein expression of genes related to milk fat and protein synthesis in heat-stressed mammary tissues (**A**) and BMECs (**B**). Results are expressed as mean ± SEM from three independent replicates. * *p* < 0.05; ** *p* < 0.01.

In order to determine whether heat stress affects milk synthesis in dairy cows, we examined the protein levels of genes related to milk fat and protein synthesis. Western blot data demonstrated that heat stress reduced protein levels of the lipid synthesis mediators FASN and SREBP1, as well as the protein synthesis marker CSN2 (Figure 3A). Meanwhile, the expression of SREBP1 showed a significant downregulation in BMECs after heat treatment at 43 °C for 2, 3 and 4 h, and CSN2 expression was remarkably reduced during 1 to 3 h of heat exposure, then began to recover and exhibited an increasing trend (Figure 3B).

### 3.4. Intracellular Reactive Oxygen Species Measurement

**Figure 4:**
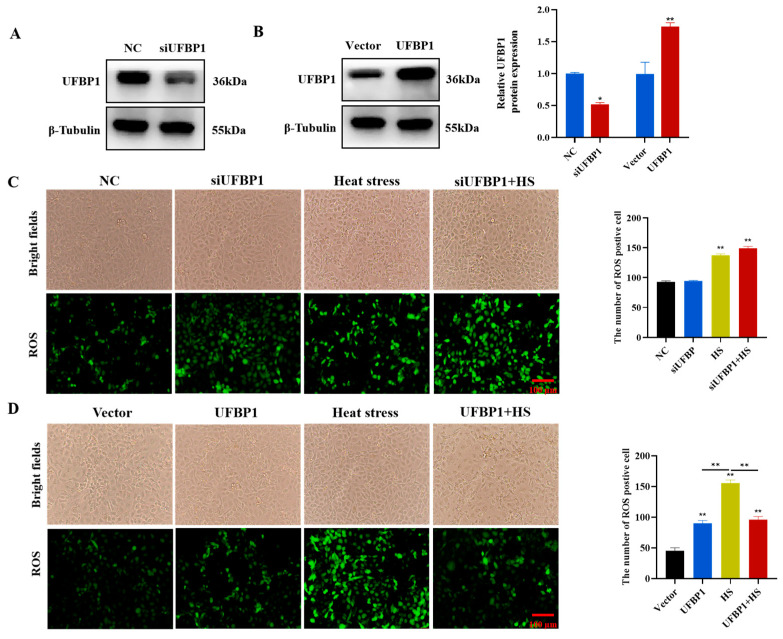
Effect of UFBP1 silencing and overexpression on ROS levels in BMECs. (**A**,**B**) Transfection efficiency of UFBP1 knockdown and overexpression. (**C**,**D**) ROS levels in UFBP1 silenced (**C**) and overexpressed (**D**) BMECs after heat treatment. Results are expressed as mean ± SEM from three independent replicates. * *p* < 0.05; ** *p* < 0.01.

ROS can serve as signaling molecules that participate in various cellular processes, including apoptosis. Thus, we explored the effects of UFBP1 on intracellular ROS production in heat-stressed BMECs. Firstly, we knocked down and upregulated the expression of UFBP1 in BMECs using small interfering RNA and overexpression plasmid. The results showed that the knockdown and overexpression levels of UFBP1 met the requirements for subsequent experiments (Figure 4A,B). The ROS results showed that the ROS levels under heat stress, UFBP1 knockdown, and their combination treatment were markedly elevated compared with the control group (Figure 4C). Conversely, overexpression of UFBP1 significantly reduced heat-induced ROS production in BMECs (Figure 4D).

### 3.5. UFBP1 Improves Heat Stress-Induced Mitochondrial Dysfunction in BMECs

**Figure 5:**
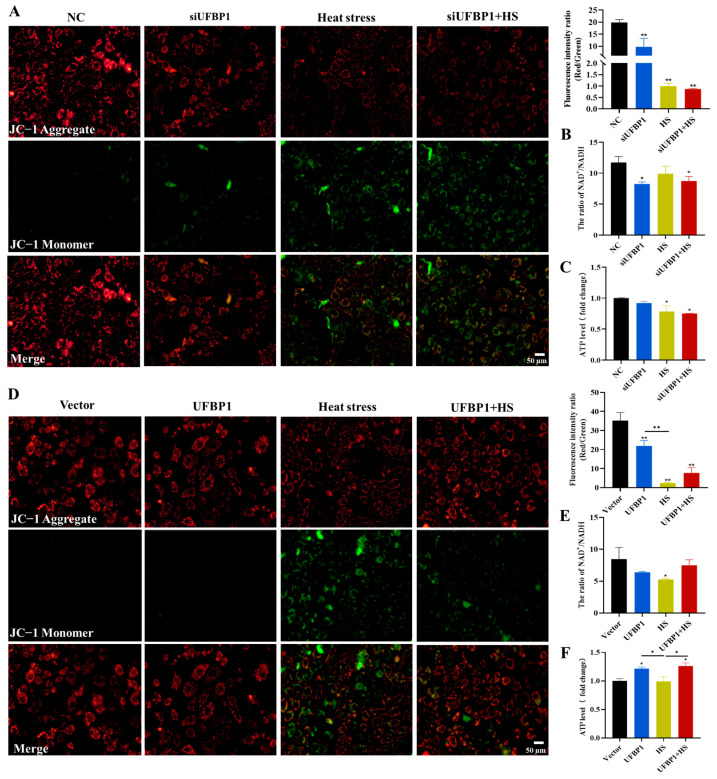
Effect of UFBP1 on mitochondrial dysfunction in heat-stressed BMECs. (**A**,**D**) JC-1 staining in UFBP1-deficient (**A**) and overexpressed (**D**) BMECs. (**B**,**E**) Intracellular NAD^+^/NADH ratio levels in UFBP1-deficient (**B**) and overexpressed (**E**) BMECs. (**C**,**F**) ATP contents in UFBP1-deficient (**C**) and overexpressed (**F**) BMECs. Results are expressed as mean ± SEM from three independent replicates. * *p* < 0.05; ** *p* < 0.01.

Considering that mitochondria are an important source of ROS, we then investigated the role of UFBP1 in mitochondrial function. JC-1 fluorescence staining revealed that the mitochondrial membrane potential (MMP), intracellular NAD^+^/NADH ratio and ATP content in BMECs was remarkably reduced by heat stress, UFBP1 knockdown and a combination treatment (Figure 5A–C). On the contrary, overexpression of UFBP1 can restore mitochondrial function, as evidenced by the increased levels of MMP, NAD^+^/NADH ratio and ATP production (Figure 5D–F).

### 3.6. UFBP1 Regulates Heat Stress-Induced Apoptosis in BMECS

**Figure 6:**
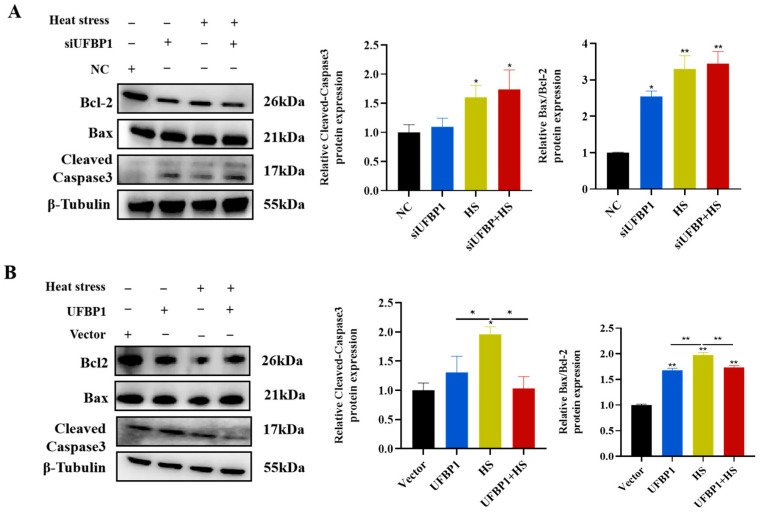
Effect of UFBP1 on heat stress-induced apoptosis in BMECs. (**A**,**B**) Western blot and quantitative evaluation of apoptotic proteins in UFBP1-depleted (**A**) and overexpressed (**B**) BMECs following heat treatment. Results are expressed as mean ± SEM from three independent replicates. * *p* < 0.05; ** *p* < 0.01.

To evaluate the effects of UFBP1 on heat stress-induced apoptosis, we downregulated and upregulated the expression of UFBP1 by transfecting UFBP1-siRNA and overexpression plasmid into BMECs. The Western blot analysis found that the protein production of apoptosis-related genes cleaved-caspase3 and Bax/Bcl-2 was significantly increased after heat treatment and UFBP1 depletion (Figure 6A). On the contrary, overexpression of UFBP1 markedly reduced the protein expression of cleaved-caspase3 and Bax/Bcl-2 in BMECs treated with heat exposure (Figure 6B), indicating that UFBP1 could effectively alleviate heat treatment-induced apoptosis.

### 3.7. UFBP1 Regulates Milk Fat and Protein Synthesis-Related Gene Expression in Heat-Stressed BMECs

**Figure 7:**
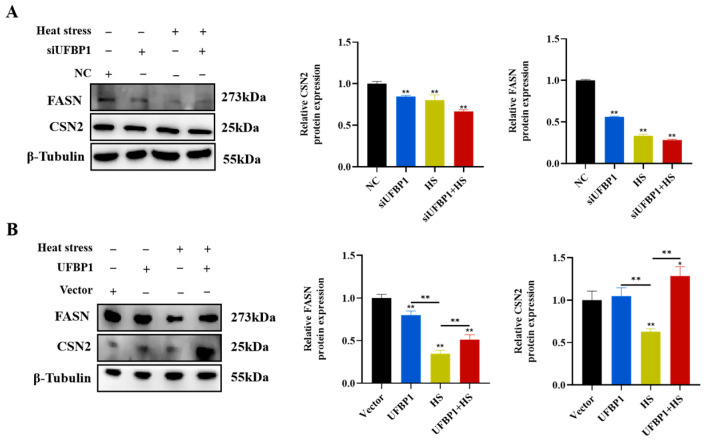
Effects of UFBP1 depletion (**A**) and overexpression (**B**) on protein expression of the genes involved in milk fat and protein synthesis. Results are expressed as mean ± SEM from three independent replicates. * *p* < 0.05; ** *p* < 0.01.

To assess whether UFBP1 affects milk synthesis during heat treatment, UFBP1 was knocked down and overexpressed, and then the milk fat and protein synthesis-related gene expression was measured. Western blot data revealed that protein levels of FASN and CSN2 were significantly decreased in heat-treated BMECs, and UFBP1 silencing further enhanced the adverse effect of heat stress on milk fat and protein biosynthesis (Figure 7A). On the other hand, compared to the heat stress group, UFBP1 overexpression considerably increased the expression of FASN and CSN2, which involved in milk fat and protein synthesis, respectively (Figure 7B).

## 4. Discussion

Heat stress in dairy cows, especially high-producing cows, is a critical catalyst of decreased lactation performance in summer due to global warming. And the reduction in lactation is closely associated with BMEC injury caused by high ambient temperatures. Our current study mainly concentrated on the regulating role of UFBP1 on heat stress-induced apoptosis in bovine mammary epithelial cells. The results indicated that UFBP1 knockdown exacerbated heat stress-induced cell apoptosis and mitochondrial dysfunction, and suppressed the expression of milk fat and protein biosynthesis-related genes; overexpression of UFBP1 effectively improved cell survival and mitochondrial function, thus upregulating the milk synthesis-related gene expression in BMECs. Through knockdown and overexpression experiments, we have validated the protective role of UFBP1 in BMECs injury under heat stress. These findings suggest that UFBP1 holds potential therapeutic value for alleviating heat stress in dairy cows.

Under heat stress conditions, there is excessive accumulation of intracellular ROS, which can cause oxidative damage to mitochondrial proteins and structures and further trigger mitochondrial dysfunction [29,30,31]. Mitochondrial dysfunction is characterized by collapsed mitochondrial membrane potential (MMP), reduced ATP synthesis, decreased NAD^+^/NADH ratio, etc. [32,33]. Bioinformatics analysis of UFBP1-regulated proteins indicated that the regulation of oxidation-reduction processes is influenced by UFBP1, and UFBP1 could regulate the production of ROS in MGC803 and AGS cells [34]. In the present study, we observed overaccumulation of ROS in UFBP1-depleted cells followed by heat stress. At the same time, UFBP1 overexpression considerably inhibited ROS production. Furthermore, heat stress-induced mitochondrial dysfunction was effectively improved by targeting UFBP1.

Mitochondria are not only the main source of intracellular ROS generation, but also play a key role in initiating and executing programmed cell death (i.e., apoptosis). When mitochondrial dysfunction occurs, the mitochondria-dependent apoptotic pathway can be triggered, thereby leading to the release of cytochrome c and the subsequent formation of apoptosome, which in turn result in the activation of downstream effector caspases (such as caspase-3), ultimately triggering apoptosis [35]. It has been ascertained that heat stress could induce the apoptosis of bovine mammary epithelium, thereby compromising milk production efficiency and mammary gland health [31,36]. In agreement with earlier findings, the present study observed that heat treatment significantly increased the activation of the apoptotic pathway. UFBP1, a key regulator of ER homoeostasis, is indispensable for ER stress-mediated cell apoptosis. Loss of UFBP1 leads to ER stress and exacerbated apoptotic cell death in various cell types, such as MCF7, HepG2, pancreatic beta and hematopoietic stem/progenitor cells [22,24]. In addition, UFBP1 has been shown to recruit ufmylation components UFL1 and CDK5RAP3 to form a large protein complex, which is involved in the maintenance of cellular homeostasis [23]. Our works, combined with recent previous studies, found that UFL1 and CDK5RAP3 could protect BMECs from apoptosis in BMECs induced by multiple stimuli, thereby facilitating cell survival [37,38]. In this research, it has been observed that UFBP1 silencing leads to an increased level of cell apoptosis in heat-stressed BMECs; overexpression of UFBP1 could effectively protect BMECs against apoptosis. The current study showed that UFBP1 exerts an anti-apoptotic effect on BMECs under heat stress, in agreement with previous reports. Collectively, these results suggest that UFBP1 attenuates heat stress-induced BMEC apoptosis by restoring mitochondrial function.

Bovine mammary epithelial cells, serving as key contributors to milk secretion, play a pivotal role in the secretory functions of mammary gland. Previous research has shown that heat stress exerts detrimental effects on BMECs. The number of BMECs decreases significantly during heat stress due to apoptosis, which ultimately inhibits the synthesis of milk components [31,39,40]. Consistent with these previous studies, we found that heat stress remarkably reduced the levels of milk fat and protein-related genes. A previous study by our team found that UFL1, the upstream gene of UFBP1 in ufmylation, could regulate the levels of genes involved in the synthesis of milk fat and protein in BMECs [41]. Therefore, UFBP1, the substrate protein of UFL1, was speculated to have the same function in regulating the levels of genes involved in milk synthesis. Similarly, we discovered that UFBP1 knockdown markedly decreased the levels of milk fat and protein-related genes, and on the contrary, overexpression of UFBP1 significantly restored the levels of those genes. However, the mechanism by which UFBP1 regulates the expression of milk fat lactation protein-related genes needs to be studied in depth.

## 5. Conclusions

In conclusion, our research demonstrated that heat stress induced cell apoptosis and decreased the levels of milk fat and protein-associated genes both in vivo and in vitro. UFBP1 alleviated heat stress-induced apoptosis in BMECs by attenuating mitochondrial dysfunction and could regulate the expression of genes related to milk fat and protein synthesis. These results indicated that UFBP1 possessed potential therapeutic value for mitigating heat stress in dairy cows, thereby providing novel theoretical insights into the mitigation of adverse thermal stress effects on livestock productivity.

## Data Availability

All data presented in this study are available on reasonable request from the corresponding author.
